# *In vitro* effects of nutraceutical treatment on human osteoarthritic chondrocytes of females of different age and weight groups

**DOI:** 10.1017/jns.2021.79

**Published:** 2021-09-24

**Authors:** Mahmoud Amr, Alia Mallah, Haneen Abusharkh, Bernard Van Wie, Arda Gozen, Juana Mendenhall, Vincent Idone, Edwin Tingstad, Nehal I. Abu-Lail

**Affiliations:** 1 Department of Biomedical Engineering and Chemical Engineering, The University of Texas at San Antonio, San Antonio, TX78249, USA; 2 Gene and Linda Voiland School of Chemical Engineering and Bioengineering, Washington State University, Pullman, WA99164-6515, USA; 3 School of Mechanical and Materials Engineering, Washington State University, Pullman, WA99164-2920, USA; 4 Department of Chemistry, Morehouse College, Atlanta, GA30314, USA; 5 Regeneron Pharmaceuticals Inc, Tarrytown, NY10591, USA; 6 Inland Orthopedic Surgery and Sports Clinic, Pullman, WA99163, USA

**Keywords:** Age, Articualr cartilage, Catechin hydrate, Nutraceuticals, Osteoarthritis, Weight, α or vitamin E, α-tocopherol, AA or vitamin C, ascorbic acid, AC, articular cartilage, BMI, body mass index, C, catechin hydrate, ECM, extracellular matrix, G, gallic acid, GAG, glycosaminoglycan, hAChs, human articular chondrocytes, MMP, metalloproteinase, NO, nitric oxide, NOS, NO Synthase, OA, osteoarthritis, TKR, total knee replacement, TNF-α, tumour necrosis alpha

## Abstract

The *in vitro* effects of four nutraceuticals, catechin hydrate, gallic acid, α-tocopherol and ascorbic acid, on the ability of human osteoarthritic chondrocytes of two female obese groups to form articular cartilage (AC) tissues and to reduce inflammation were investigated. Group 1 represented thirteen females in the 50–69 years old range, an average weight of 100 kg and an average body mass index (BMI) of 34⋅06 kg/m^2^. Group 2 was constituted of three females in the 70–80 years old range, an average weight of 75 kg and an average BMI of 31⋅43 kg/m^2^. The efficacy of nutraceuticals was assessed in monolayer cultures using histological, colorimetric and mRNA gene expression analyses. AC engineered tissues of group 1 produced less total collagen and COL2A1 (38-fold), and higher COL10A1 (2⋅7-fold), MMP13 (50-fold) and NOS2 (15-fold) mRNA levels than those of group 2. In comparison, engineered tissues of group 1 had a significant decrease in NO levels from day 1 to day 21 (2⋅6-fold), as well as higher mRNA levels of FOXO1 (2-fold) and TNFAIP6 (16-fold) compared to group 2. Catechin hydrate decreased NO levels significantly in group 1 (1⋅5-fold) while increasing NO levels significantly in group 2 (3⋅8-fold). No differences from the negative control were observed in the presence of other nutraceuticals for either group. In conclusion, engineered tissues of the younger but heavier patients responded better to nutraceuticals than those from the older but leaner study participants. Finally, cells of group 2 formed better AC tissues with less inflammation and better extracellular matrix than cells of group 1.

## Introduction

Articular cartilage (AC) is a tissue that surrounds moving joints, providing lubrication and serves as a load-bearing tissue in joints such as the knee. AC has a low cellular density of chondrocytes embedded in a highly organised extracellular matrix (ECM) composed mainly of collagen II and glycosaminoglycans (GAGs)^(1)^. The degradation of ECM due to injury^(2)^, genetics^(3)^, obesity^(4)^ and aging^(5)^ results in osteoarthritis (OA). OA affects more than 30 million people in the USA, causing a huge financial burden of an estimated $137 billion annually^(6)^. OA worsens the quality of life, often progresses to disability. AC has a very limited ability to self-heal due to its avascularand alymphatic nature. The aneural nature of tissue results in lack of pain sensation and makes it difficult to detect OA in its early stages. OA has no approved disease-modifying drugs that can result in tissue healing. It is largely managed for symptomatic pain relief using pain killers, anti-inflammatory injections and intra-articular injections of lubricating polymers such as hyaluronic acid. Eventually and as the disease progresses, total knee replacement (TKR) surgery is needed^(7)^.

Many factors affect OA such as sex, aging and obesity^(4,5)^. In 2018, 49⋅6 % of self-reported OA patients were in ages above 65 compared to 29⋅3 % in ages 45–64 years old, and 30⋅3 % of the OA patients aged 18 and above were females compared to 22⋅9 % males^(8)^. With aging, changes in the joint can be systemic such as loss of activity and balance, as well as increased cell senescence, the exact pathway of which is unknown^(9)^. The first National Health and Nutrition Examination Survey has shown that obese females were four times at higher risk of knee OA than non-obese females^(10)^. Studies as well related the high levels of adipokines to an increase in MMP13 levels in obese patients^(11)^. Finally, increase in mechanical loading on the joints leads to further destruction of degraded joints^(12)^.

The exact underlying molecular mechanisms behind the evolution of OA are not very well understood. However, a great deal of effort has been directed towards the study of inflammation of the joint marked by oxidative stress in OA^(13)^. In early stages of OA, chondrocytes express degradative enzymes like metalloproteinase (MMP)^(14)^ that are exacerbated by the production of inflammatory cytokines such as tumour necrosis alpha (TNF-α)^(15)^ and interleukins (IL)^(16)^. The increase in MMP levels leads to further degradation of the ECM denoted by a reduction in collagen II and an increase in chondrocytes' hypertrophy with higher levels of collagen I and collagen X expressed^(17)^. As such, controlling inflammation in OA is a step towards the control of ECM degradation.

Patients resort to natural and home remedies to alleviate the pain associated with OA and to reduce inflammation^(18)^. Nutraceuticals are naturally occurring anti-inflammatory and antioxidant chemicals available in everyday diets and sometimes are taken as supplements due to their general safety^(19)^. As antioxidants, nutraceuticals possess a scavenging ability of reactive oxygen species (ROS) . As such, they restore the balance in ROS levels and alleviate the oxidative stress. Nitric oxide (NO) is a major ROS, produced by NO synthase (NOS) in the event of inflammation, which acts as a mediator in pathophysiological processes in cells with an increase in NOS gene expression; indicating more inflammation in OA joints^(20)^. The exact mechanism of oxygen scavenging is nutraceutical-dependent. Because nutraceuticals are not regulated by the food and drug administration, many options are available over-the-counter. The responsibility to test the efficacy of these supplements towards OA relies upon the scientific community.

The lack of controlled studies investigating the underlying interconnected mechanisms of aging and excessive weight in OA in response to nutraceutical treatments motivated the present study. We studied the effect of four nutraceutical treatments: catechin hydrate (C), an active ingredient in green tea; gallic acid (G), available in gallnut; α-tocopherol (α or vitamin E), available in mixed nuts; ascorbic acid (AA or vitamin C), available in citrus fruits on inflammation reduction in chondrocytes obtained from obese female patients of two groups (group 1: 50–69 years old, an average weight of 100 kg and an average body mass index (BMI) of 34⋅06 kg/m^2^) and (group 2: 70–80 years old, an average weight of 75 kg and an average BMI of 31⋅43 kg/m^2^). The effects of nutraceuticals on chondrogenic enhancements were also assessed. Implications for the use of nutraceuticals as complementary dietary components for patients with OA are discussed.

## Materials and methods

The following materials were acquired from Life Technologies Corp (Waltham, MA, USA): Alamar blue, collagenase type I, Dulbecco's modified Eagle's medium (DMEM), fetal bovine serum (FBS), fungizone, MagMAX™-96 for microarrays total RNA isolation kit, penicillin/streptomycin (Pen/Strep), and quant-iT PicoGreen kit and SuperScript® VILO™ master mix. The following materials were acquired from Millipore Sigma (St. Louis, MO, USA): α, AA, Bouin's fixative, catechin hydrate, chloroform, dimethyl sulphoxide (DMSO), ethanol, ethylenediaminetetraacetic acid (EDTA) disodium salt , G, iso-propanol, L-cysteine hydrochloric acid (HCl), Masson's trichrome kit, phosphoric acid, rat collagen I, sodium acetate, sodium nitrite, steriflip filters, sulphanilamide, toluidine blue, TRIzol, and trypan blue. The insoluble collagen kit was obtained from Biocolor Ltd (UK), phosphate-buffered saline (PBS) was obtained from GE Healthcare Life Sciences (Marlborough, MA, USA) and the TaqMan® gene expression master mix was obtained from Applied Biosystems (Grand Island, NY).

### Isolation of human articular chondrocytes

Adult human articular chondrocytes (hAChs) were obtained from sixteen osteoarthritic female, Caucasian patients with stage 4 OA (International Cartilage Repair Society Scale) who underwent TKR surgeries. AC tissues discarded during surgeries were provided by Dr. Edwin Tingstad. The study was performed on two groups: Group 1: Age range 50–69 years, average age, weight and BMI: 63 years, 100 kg and 34⋅06 kg/m^2^, respectively, *n* 13; group 2: Age range: 70–80 years, average age, weight and BMI: 76 years, 75 kg and 31⋅43 kg/m^2^, respectively, *n* 3. The mean weights between the two groups were statistically different (Supplementary Figure S1), while the mean BMIs were not different. Since we can't control patients who undergo TKR, we selected our samples from those who did the surgery over a year. When it came to group 2, we were only able to collect samples from three female patients with the criteria identified earlier. We can speculate that the limited number of patients in group 2 who underwent TKR during the year was in part due to elderly patients having additional medical issues that prevent them from doing TKR surgeries or possibly due to lack of elderly population in Pullman, WA which is a small university town.

AC tissues were dissected into pieces from seemingly less inflamed regions. Tissues were washed three times with dissection medium (PBS containing 1 % Pen/Strep and 1 % Fungizone). AC tissues were digested overnight at 37°C and 125 rpm using a digestion medium (DMEM/Ham's F-12 containing 0⋅1 % collagenase yype I, 2 % FBS, 2 % Pen/Strep and 1 % fungizone) and then filtered through 40 μm Steriflip filters. The enzyme was deactivated by diluting the digested tissue with an expansion medium (DMEM/Ham's F-12 containing 10 % FBS, 1 % Pen/Strep and 1 % Fungizone), followed by three washes with DMEM and 10 min centrifugations at 1500 rpm. Cells were counted using 0⋅4 % trypan blue and then suspended in freezing medium (90 % expansion medium and 10 % DMSO) at a density of 1 million cells/ml in 2 ml freezing vials and frozen in a −84°C freezer until use.

The present study was conducted according to the guidelines laid down in the Declaration of Helsinki and all procedures involving human patients were approved by the Washington State University Institutional Review Board (IRB) #17087. Written informed consents were obtained from all patients.

### Cell culture

Vials of frozen cells were thawed. Cells were washed three times with the expansion medium after thawing to get rid of residual freezing medium and counted with 0⋅4 % trypan blue. Cells were cultured at a seeding density of 16 million cells/ml. Cultures were incubated in a humidified CO_2_ incubator at 37°C for 21 days with the medium changed every other day. To study the effect of the four nutraceuticals (C, G, α and AA) on OA hAChs, four expansion media were prepared with a final concentration of 50 μM of each nutraceutical as well as a negative control (NC) medium that had no nutraceuticals. To perform experiments designed, OA hAChs were pooled from different donors to yield a representative mixture of chondrocytes for the two age and weight groups investigated. This was done for several reasons. First, pooling of cells is a common practice to reduce variability amongst donors^(21–24)^. Secondly, OA hAChs dedifferentiate upon expansion; limiting the ability to increase cell numbers to cell densities desired *via* expansion^(25)^. Finally, we wanted to investigate responses of primary cells and not expanded cells. After pooling, OA hACh suspensions were seeded at random in 24-well plates and were investigated in technical triplicates.

### Cell viability

The Alamar blue test was performed to assess the viability of the cultured hAChs at day 21, according to manufacturers’ protocol. In short, Alamar blue reagent was added to medium to make a 10 % v/v and incubated at 37°C and 5 % CO_2_ for 4 hours. The absorbance was measured against a blank of medium and Alamar blue at a wavelength of 570 nm using Cytation 5 Multiplate Reader (BioTek, Winooski, VT, USA). Samples were run in triplicates. The value of the measured absorbance is an indication of cell viability as only living cells can reduce the active ingredient.

### Nitric oxide assay

To determine the NO levels in culture medium, Greiss reaction was employed as described previously with some modifications^(26)^. The reduction of NO^3−^ to NO^2−^ was detected spectrophotometrically by adding Griess reagents [0⋅1 % N-(1-Napthyl)ethylenediamine dihydrochloride (NED) in nanopure water (resistivity: 18⋅2 mΩ) and 1 % sulphanilamide in 5 % phosphoric acid] to the medium. A standard was prepared using a stock solution of 0⋅1 M sodium nitrite in nanopure water. Sulphanilamide and NED solutions were equilibrated to room temperature for 30 minutes before use. Volumes of 50 μl from media were placed in a 96-well plate in triplicate and 50 μl of sulphanilamide was added to each well. The mixture was incubated for 10 min at room temperature in the dark to which 50 μl of NED solution was added to each well. The new mixture was incubated for 10 more minutes at room temperature in the dark. The absorbance of the final mixture was then measured at 543 nm using a Cytation 5 Multiplate Reader.

### Biochemical analyses of total collagen and DNA

Total collagen produced was measured using an insoluble collagen kit. A modified version of the manufacturer's protocol was used. Briefly, 400 μl/well of the dye was added and the plate was shaken for 30 minutes on an orbital shaker. The dye was then removed and 400 μl of acid/salt wash was added to remove the unbound dye. The mixture was incubated on the shaker for 10 minutes after which 400 μl of dye dissociation reagent was added to the wells to dissolve the bound dye. The dye was then taken to a 96-well plate and absorbance was measured at 550 nm using Cytation 5 Multiplate Reader. DNA's quantification was performed on the same samples after collagen's quantification, dye's removal and washing steps.

To determine the amount of DNA in the samples, Quant-iT PicoGreen kit was used according to the manufacturer's protocol. Briefly, cells were digested overnight at 60°C in 0⋅1 mg/ml papain digestion medium (0⋅2 M sodium phosphate buffer containing 8 mg/ml sodium acetate, 4 mg/ml EDTA disodium salt and 0⋅8 mg/ml L-cysteine HCl). 12⋅5 μl of the samples were added to a 96-well plate. To that, 87⋅5 μl of 1×TE buffer was added. After that, a 100 μl of 1:1000 diluted PicoGreen reagent was added. The well plate was incubated in the Cytation 5 Multiplate Reader with gentle shaking for 3 minutes after which the fluorescence was measured at an excitation of 480 nm and an emission of 520 nm.

### Histology

Qualitatively, stainings of total collagen and total GAG were done using aniline blue (Masson's Trichrome) and Toluidine Blue, respectively. For both tests, on day 21, cells were fixed using Bouin's fixative for 15 minutes at 56°C. The fixative was washed with deionised (DI) water until its yellow colour cleared. For total collagen, the staining was done according to the manufacturer's protocol. Briefly, aniline blue was added to the wells for 10 minutes. The dye was removed and 1 % acetic acid was added to the wells to differentiate the colour. GAG's staining was carried out as previously described^(27)^. Briefly, the wells were covered by (0⋅1 % toluidine blue in 5 % acetic acid) for 5 minutes, the dye was washed using DI water. Histological images of the stained cultures were captured using a Nikon-inverted microscope (Nikon Corporation, Tokyo, Japan) at 10X magnification.

### mRNA isolation and analysis

To quantify the amount of mRNA expressed in the cells, quantitative real-time polymerase chain reaction was used^(28)^. Briefly, total mRNA was isolated using TRIzol™. Chloroform was used to achieve phase separation between the aqueous and organic phases. The mRNA-containing aqueous phase was purified using MagMAX™-96 for microarrays total RNA isolation kit as per manufacturer's protocol. Total mRNA (up to 2⋅5 μg) was reverse transcribed to core DNA (cDNA) using SuperScript® VILO™ master mix. cDNA was amplified with TaqMan® gene expression master mix on an ABI 7900HT sequence detection system (Applied Biosystems, Grand Island, NY) and probes that are specific for the housekeeping gene human β-actin, COL2A1, COL10A1, ACAN, SOX9, FOXO1, MMP13, BMP2, TNFAIP6 and NOS2 were used. Relative gene expression was calculated using the ΔΔC_T_ method previously described^(29)^, and fold differences were expressed as 2^−ΔΔC^_T_.

### Statistical analysis

To study the significance of differences between the two groups, two-way ANOVA was performed with multiple comparisons using Tukey's test. Finally, Grubb's test was performed to remove outliers from the data^(30)^. GraphPad Prism (GraphPad Software, San Diego, CA) was used.

## Results

### Cell viability

Chondrocytes derived from both groups were viable at day 21 with no significant differences in viability between treatment groups or between the two groups (two-way ANOVA) (Supplementary Figure S2).

### ECM formation

**Fig. 1.**
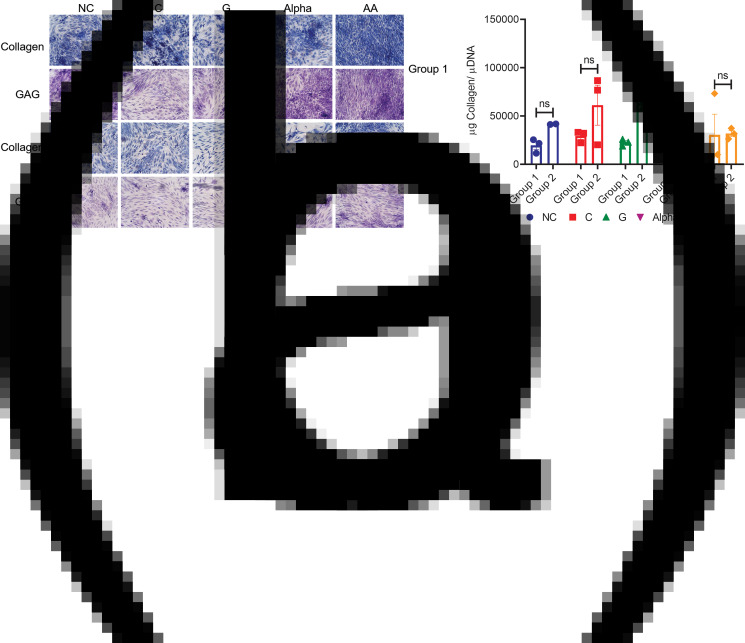
(a) Representative histological images of total collagen (Aniline Blue Staining) and GAG (Toluidine Blue Staining) for both groups (Objective: 10X), scale bar is shown in Supplementary Figure S3 (*n=*3). (b) Normalised total collagen per DNA measured at day 21 for chondrocytes of both groups (mean ± sem, *n=*3), not significant (ns): *P* > 0⋅05 and significant:*P < 0⋅05.

Qualitatively, chondrocytes from both groups produced ECM proteins containing collagen and GAG as indicated by 21-days cultures (representative images in Fig. 1(a)). It has to be noted that the density of stained tissues in these images is not stoichiometrically correlated to contents of collagen and GAG in tissues imaged. As such, darker staining of GAG or collagen in images does not necessarily indicate a higher content of the markers imaged in the tissues. To quantify collagen's and GAG's formation stoichiometrically from all sample content, colorimetric assays were used. The present results indicated that, while G showed the only statistically significant increase in collagen by 3⋅2-fold (Fig. 1(b), two-way ANOVA), the general trend of increasing values of 1⋅7, 2⋅1, 2⋅3 and 1⋅0-fold was observed for NC, C, α and AA treatments, respectively.

### Nitric oxide levels

**Fig. 2.**
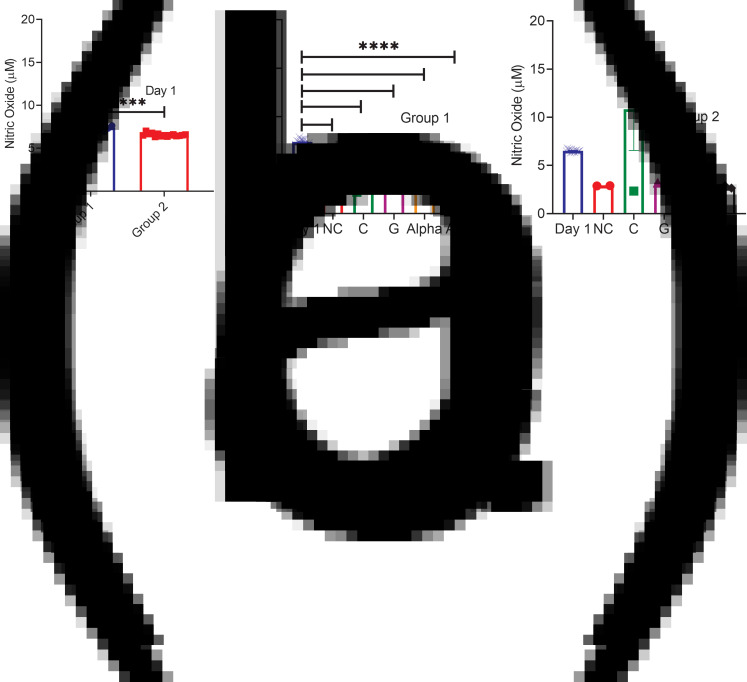
(a) NO levels of both groups at day 1 (mean ± sem, *n* = 15). Prior to any treatment at day 0, the NO content was averaged for all 15 samples representing the technical replicate for the negative control and that of each of the four nutraceuticals (4 × 3) investigated as they all come from the same pool prior to randomization of cells in wells. (b) and (c) NO levels of both groups measured at day 21 *v.* day 1, respectively (mean ± sem, *n* = 3). *P < 0⋅05, **P < 0⋅01, ****P < 0⋅0001.

After culturing for 21 days, NO dropped for all studies, significantly for group 1 by an average of 2⋅6-fold (Fig. 2(a)). Though not significant, the very similar collective average drop of 2⋅3-fold for all but C corroborated the group 1 data. While adding nutraceuticals showed average drops from NC of 1⋅3-fold and 1⋅4-fold for groups 1 and 2 (except for C), respectively, only C tretament in group 1 showed a significant 1⋅5-fold drop from NC while increasing NO levels by 3⋅8-fold for group 2 (Fig. 2(b) and 2(c)).

### mRNA relative gene expression of the ECM proteins (COL2A1, COL10A1 and ACAN)

**Fig. 3.**
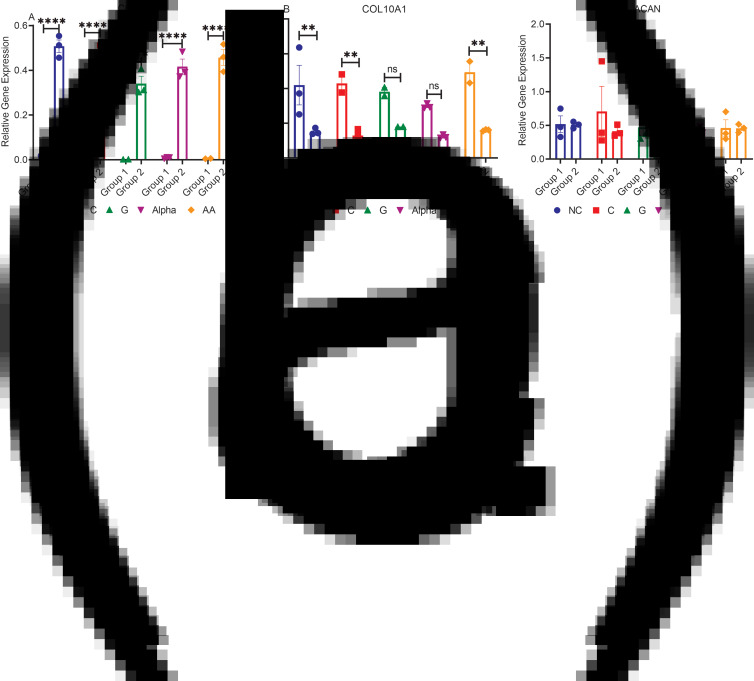
mRNA relative gene expressions of (a) COL2A1, (b) COL10A1 and (c) ACAN for both groups (mean ± sem, *n*3): **P < 0⋅01, ****P < 0⋅0001 and ns *P* > 0⋅05.

In characterising the mRNA genes responsible for translating the main ECM components, a significant impact on participant weight is noted. We studied the chondrogenic genes COL2A1 and ACAN and the hypertrophic gene COL10A1. The present results indicated that group 2 expressed significantly more chondrogenic collagen II mRNA (Fig. 3(a)) (38-fold), which coincides with enhanced collagen production inferred by the data in Fig. 1(b). Significantly less osteogenic collagen X mRNA (Fig. 3(b)) (2⋅7-fold) was produced compared to group 1. Both groups (Fig. 3(c)) expressed similar levels of ACAN with no significant differences observed among treatments nor compared to NC.

### mRNA relative gene expression of the regulatory proteins (BMP-2, FOXO1 and SOX9)

**Fig. 4.**
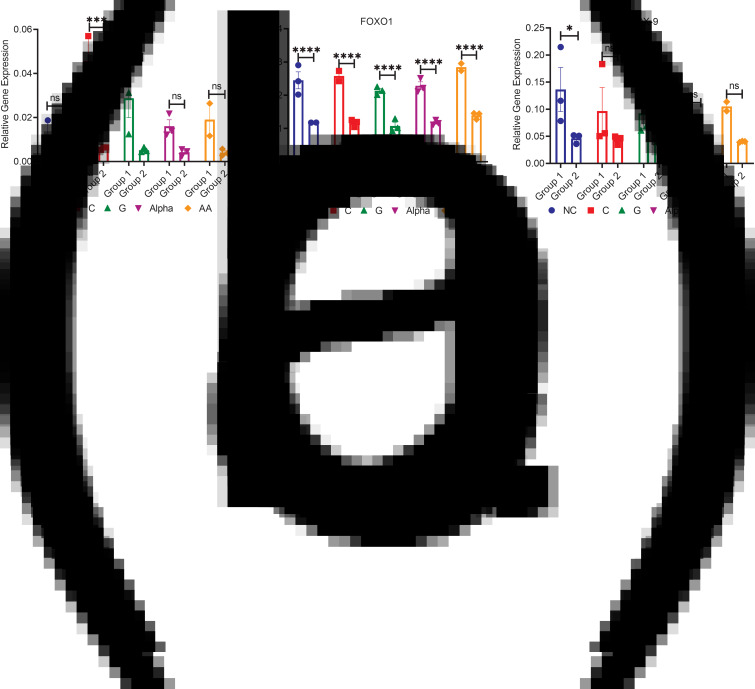
mRNA relative gene expressions of (a) BMP-2, (b) FOXO1 and (c) SOX9 for both groups (mean ± sem, *n*3). *P < 0⋅05, **P < 0⋅01, ***P < 0⋅001, ****P < 0⋅0001 and ns *P* > 0⋅05.

The relative gene expressions of three important regulatory proteins of AC homoeostasis and function were assessed. These are the bone morphogenetic protein-2, forkhead-box O1 and sex-determining region Y box-9 (BMP-2, FOXO1 and SOX9). BMP-2 relative gene expression was higher in group 1 with significance from NC detected only in groups treated with C and G (5-fold)(Fig. 4(a)). No significant differences were detected in group 2 between treatments and NC (Fig. 4(a)). Group 1 expressed significantly more FOXO1 than group 2 for all treatments (2-fold), with no significant differences between treatments and NC in both groups (Fig. 4(b)). The levels of SOX9 mRNA relative gene expression were not significantly different between the two groups except for NC (Fig. 4(C)). Group 1 NC were significantly higher than group 2 NC (*P* < 0⋅05) (Fig. 4 (C)). No significant differences were detected between treatments and NC for both groups (Fig. 4(C).

### mRNA relative gene expression of inflammation-related markers (NOS2, MMP13 and TNFAIP6)

**Fig. 5.**
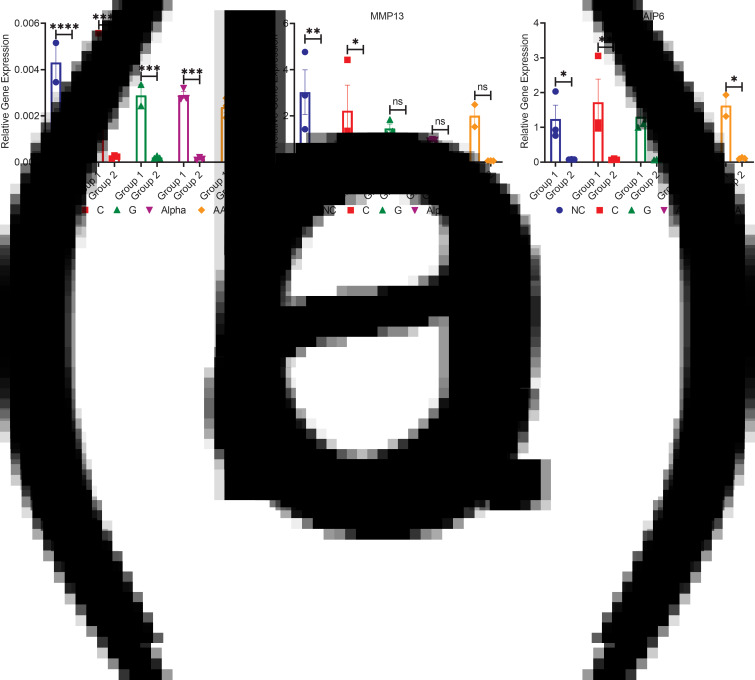
mRNA relative gene expression of (a) NOS2, (b) MMP13 and (c) TNFAIP6 (mean ± sem, *n*3) for both groups. *P < 0⋅05, **P < 0⋅01, ***P < 0⋅001, ****P < 0⋅0001 and ns *P* > 0⋅05.

Group 1 expressed significantly more NOS genes than group 2 for all treatments (15-fold), with no significant differences between nutraceutical treatments for either group (Fig. 5(a)). These results confirm our NOS assay results measured with the Griess reaction (Fig. 2). Here, the present results indicated that MMP13 levels for group 1 were higher than those of group 2 (50-fold), with significance only between NC- and C-treated chondrocytes when comparing the two groups (Fig. 5(b)). Finally, group 1 expressed significantly more TNFAIP6 than group 2 (16-fold) (Fig. 5(c)). This was true except for α-treated chondrocytes in which differences were insignificant between both groups. No significance was detected between treatments and NC (Fig. 5(c)).

## Discussion

**Fig. 6.**
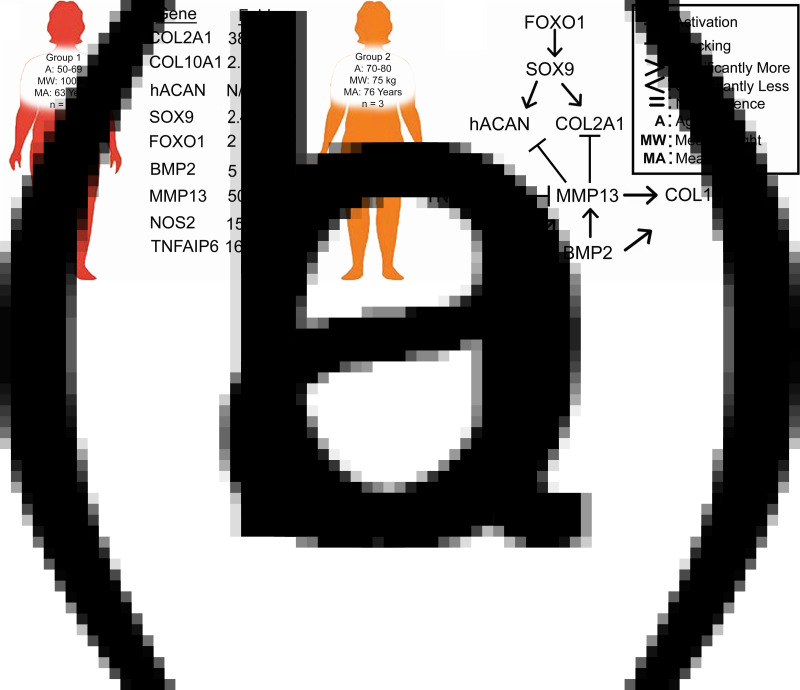
(a) Summary of gene expression differences found between the two groups investigated. (b) Complex interplay between key markers of AC homoeostasis as described in the literature.

The correlation between ECM markers, NO and gene expression in the present study provides insight towards understanding age and weight-dependent cellular responses to nutraceutical treatments of OA chondrocytes. Our main findings are summarised in Fig. 6(a). Briefly, we found no significant differences in response to nutraceutical treatments when compared to NC in both groups. However, we found that female patients in group 2 who were older yet leaner showed an inherently better phenotype and less inflammation than those of younger and more obese female patients of group 1 (Fig. 6(a)).

As we age, our body's natural ability to regenerate declines and we become prone to diseases. When it comes to OA, the changes can be at the whole joint level or at the tissue level. At the joint's level, changes such as weakened muscles or muscle loss, meniscus degeneration and bone density decrease are observed^(31)^. At the tissue's level, changes such as chondrocytes' senescence as indicated by telomere shortening^(32)^ and the formation of advanced glycation products (AGE) are evident. The creation of AGE leads to a decrease in mechanical properties of AC^(33)^. Additionally, with increasing age, chondrocytes lose their ability to respond to simulation by growth factors such as insulin-like growth factor 1 (TGF1) and transforming growth factor β 1 (TGF-β1)^(34)^. Here, group 2 NO levels dropped from day 1 to day 21 but not significantly. However, group 1 and despite their NO levels being higher on day 1 than group 2 experienced a significant drop in NO by day 21. Note that no differences appear between nutraceutical treatments and NC except for the C treatment. Similar results were observed previously by Bharrhan *et al.*, where they found that C reduced the NO levels in alcohol-induced liver injury^(35)^. It was also observed that C regulated the levels of NO production by downregulating the inducible (iNOS) via inhibition of nuclear factor kappa B^(36)^. Additionally, Kaur *et al.* showed *in vitro* that C-loaded nanoparticles reduced the NO levels by competing with oxygen to prevent the formation of nitrites tested by the Griess reaction^(37)^. Interestingly, C affected group 2 in an ooposite manner to group 1, which requires further investigation. Obesity is regarded as a state of low-grade systemic inflammation, where high levels of tumour necrosis factor-α (TNF-α), interlukin-6 (IL-6) and leptin have been observed in obese compared to normal adipocytes. This increase in adipocytes' inflammation can contribute to the worsening of OA symptoms making it a comorbidity or a risk factor^(38)^. It has also been shown that weight loss leads to a decrease in inflammatory markers such as TNF-α and IL-6 and an increase in anti-inflammatory markers such as adiponectin^(38)^. Pallu *et al.* found that chondrocytes derived from obese patients treated with leptin showed an overexpression of MMP13 suggesting a direct correlation between obesity and OA^(39)^. The worsening of OA in obese patients could also be attributed to excessive joint loading shown to increase levels of matrix-degrading enzymes^(12)^.

Among the undesired phenotypes found for group 1 in comparison to group 2 were the lower levels of COL2A1 as well as the higher levels of NOS2 and MMP13 mRNA. Van den Berg *et al.* reported that iNOS knockout in mice made them resistant to experimental OA^(40)^. Murrell *et al.* found that the inhibition of NO produced by iNOS through TNF-α and IL-1β inhibited MMP activity^(41)^. In healthy mammalian cells, the MMP enzyme family plays a critical role in matrix remodelling like in wound healing. MMPs help in the embryonic development of the cartilage-bone interface and they are involved in tissue turnover in adult cartilage^(42)^. However, the upregulation of MMPs, especially MMP13, has been linked to OA's progression and cartilage degradation^(43)^. MMP13 degrades the ECM matrix by degrading both collagen II and aggrecan (Fig. 5(b))^(7)^.

We further observed that for group 1, levels of BMP2 were higher than group 2, accompanied by higher levels of the hypertrophic COL10A1 (Fig. 3(b)). We also observed a duality in SOX9 action in chondrocytes as both groups had similar levels of SOX9, yet group 1 had higher COL10A1 while group 2 had higher COL2A1 mRNA levels. BMP-2 and SOX9 are two important proteins in controlling chondrocytes' differentiation. BMP-2 is a regulatory protein that is essential for chondrocytes' functionality. It acts however as a two-edged sword promoting chondrocytes' synthesis of ECM but also leading to terminal differentiation when accompanied by other degradative enzymes from the MMP enzyme family such as MMP13^(44)^. The latter explains what we have observed with group 1 (Figs. 6(a) and (b)). SOX9 is a DNA-binding protein that plays a key role in early developmental stages of chondrocytes^(45)^. However, the function of SOX9 is time-dependent; continued expression of SOX9 in differentiated chondrocytes is essential for hypertrophy and survival, with latter inactivation in round chondrocytes resulting in COL2A1 inhibition or under regulation, while flat chondrocytes mature without hypertrophy that leads to apoptosis^(46)^.

Furthermore, the present results indicated that group 1 expressed more FOXO1 and TNFAIP6 than group 2 (Fig. 6(a)). These results suggest that cells of group 1 are responding to nutraceutical tretament by reducing inflammation and attempting repair of the damaged tissue. FOXO transcription factors have gained a lot of interest in recent years when it comes to OA^(47)^. FOXO family has been shown to be upregulated in the inflammatory environments and have a chondroprotective role through regulating stress-related, cell growth, and survival genes and by modulating autophagy^(48)^. FOXO1 is necessary for SOX9 gene expression as well as for cell cycle arrest in chondrogenic differentiation via the TGF-β1 pathway (Fig. 6(b)^(49)^. It regulates the gene expression of ACAN and COL2A1 (Fig. 6(b))^(50)^. TNFAIP6 is an anti-inflammatory protein that is induced by TNF-α cytokine. TNFAIP6 has a hyaluronan-binding domain that serves a role in ECM's stability and cellular migration. Overexpression of TNFAIP6 has been found in patients suffering from knee OA^(51)^. TNFAIP forms a complex with inter-α-inhibitor which is a protease inhibitor which in turn leads to inhibition of MMPs^(52)^.

The differences observed in gene expression between NC and day 21 cultures for both groups as a function of nutraceutical treatment are summarised in Supplementary Table S1 which may provide insights that can be used in future studies since statistical significance was not detected. The nutraceuticals used did not protect against hypertrophy as the levels of COL2A1 and ACAN have been reduced compared to NC, while the levels of BMP-2 increased for all treatments (Fig. 6(a)). However, all nutraceuticals for both groups reduced NOS and MMP13 levels compared to NC. This reduction suggests that nutraceutical treatment may help alleviate the inflammation and reduce matrix degradation. However, such positive effects are not sufficient to counteract hypertrophy which could have been manifested due to two-dimensional (2D) culture dedifferentiation^(53)^. Cells treated with AA seemed to have a more hypertrophic phenotype compared to cells treated with other nutraceuticals as evident from the reduced levels of collagen II and aggrecan, and increased level of collagen X. This could be due to the dual functionality of AA as an osteogenic and a chondrogenic inducer ^(54)^. All nutraceuticals led to an increase in the chondroprotective TNFAIP6-gene expression. Chondrocytes of group 2 responded well to C and AA treatment, as they have caused an increase in TNFAIP6 and a decrease in NOS2 and subsequently a decrease in MMP13. Akolkar *et al.* noted that AA can act as an antioxidant by reducing NO levels *via* downregulating iNOS and endothelial NOS in doxorubicin-induced inflammation in cardiac cells^(55)^. Similar effect for C has been observed where it downregulated iNOS levels in focal cerebral ischaemia^(36)^. These results suggest a chondroprotective role for AA and C, with C significantly decreasing NO levels in cells of group 1.

In summary, the present results showed that chondrocytes of elder and leaner females (group 2) showed inherently better phenotype and less inflammation than younger and heavier females (group 1) after 21-days culture *in vitro* by expressing higher COL2A1 and less MMP13, TNFAIP6 and NOS2. These findings stress the importance of weight management to help combat the progression and worsening of OA. However, chondrocytes of group 1 responded better to nutraceuticals compared to group 2 by expressing genes for anti-inflammatory proteins. C decreased NO levels in group 1 and increased NO levels in group 2 significantly, which suggests an age-dependent effect of C. Trends in nutraceutical treatment show that all nutraceuticals led to a reduction in gene expressions of NOS2 and MMP13 and an increase in the chondroprotective protein TNFAIP6's gene expression in group 1. The nutraceuticals did not protect against hypertrophy and dedifferentiation. Finally, our findings suggest the importance of three-dimensional (3D) culture to ensure a proper chondrogenic phenotype while alleviating inflammation, which may in turn help improve AC regeneration. For future work, investigating a full panel of inflammatory proteins could provide a better understanding of the mechanisms presented in the present paper.

## Supplementary material

For supplementary material accompanying this paper visit http://dx.doi.org/10.1017/jns.2021.79. click here to view supplementary material
