# Supplementary material for: In vitro effects of nutraceutical treatment on human osteoarthritic chondrocytes of females of different age and weight groups
Source: J Nutr Sci. 2021 Sep 24;10:e82. doi: 10.1017/jns.2021.79 (PMC8477349; doi:10.1017/jns.2021.79)
Supplement: Supplementary file 1 [file S2048679021000793sup001.docx]

**Supporting Information for**

**The *in vitro* Effects of Nutraceutical-Treatment on Human Osteoarthritic Chondrocytes of Females of Different Age and Weight Groups**

Mahmoud Amr^1^, M.Sc., Alia Mallah^1^, B.Sc., Haneen Abu-Sharkh^2^, B.Sc., Bernard Van Wie^2^, Ph.D., Arda Gozen^3^, Ph.D., Juana Mendenhall^4^, Ph.D., Vincent Idone^5^, Ph.D., Edwin Tingstad^6^, MD, and Nehal I. Abu-Lail^1, *^, Ph.D.

1) Department of Biomedical Engineering and Chemical Engineering, The University of Texas at San Antonio, San Antonio, Texas, 78249, 2) Gene and Linda Voiland School of Chemical Engineering and Bioengineering, Washington State University, Pullman, Washington, 99164, 3) Department of Mechanical Engineering, Washington State University, Pullman, Washington, 99164, 4) Department of Chemistry, Morehouse College, Atlanta, Georgia, 30314, 5) Regeneron Pharmaceuticals Inc, Tarrytown, New York, 10591, and 6) Inland Orthopedic Surgery and Sports Clinic, Pullman, Washington, 99163.

**Running head title:** Nutraceuticals and osteoarthritis

*Corresponding Author:

Nehal I. Abu-Lail, One UTSA Circle, Department of Biomedical Engineering and Chemical Engineering, The University of Texas at San Antonio, San Antonio, Texas 78249

email: [nehal.abu-lail@utsa.edu](mailto:nehal.abu-lail@utsa.edu)

This work was supported in part by an NSF GOALI Grant CBET-1606226.

**Conflict of Interest:** All authors declare no conflict of interest

No reprints are requested

**S1. Differences in the weight of the two groups investigated**

Figure S1 shows the difference in weight between group 1 and group 2. Group 1: age 50 – 69 years, average age: 63 years, average weight: 100 kg, average body mass index (BMI) 34.06 kg/m^2^, n=13. Group 2: age: 70 – 80 years, average age: 76 years, average weight: 75 kg, average BMI 31.43 kg/m^2^, n=3. The average weight of group 1 was significantly more than group 2 (P < 0.05), statistical differences were tested using unpaired t-test.


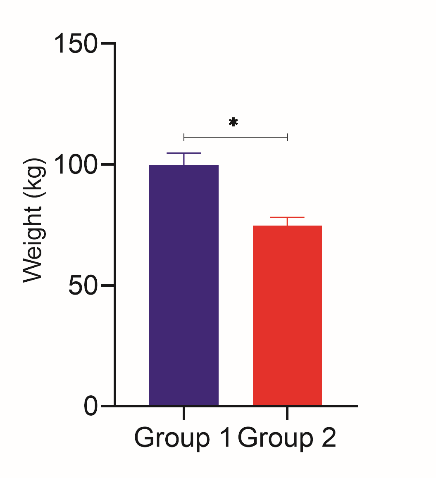


**Figure S1.** Weights of group 1 and group 2 (Mean ± SEM) (group 1, n=13; group 2, n=3), * P<0.05.

**S2. Viability of cells as a function of nutraceutical treatment**

Chondrocytes derived from both groups were viable at day 21 of culture with no significant differences in viability between treatment groups or between age groups.


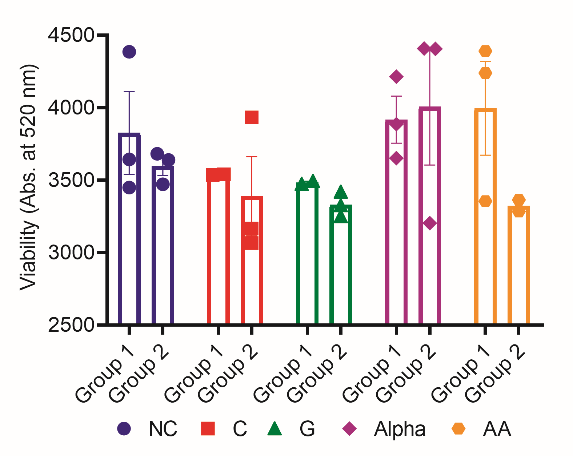


**Figure S2.** Viability of cultured chondrocytes at day 21 for both groups as a function of nutraceutical treatments. (Mean ± SEM) (n = 3). No statistical differences among age groups or nutraceutical treatments were observed.

**S3. A representative image of glycosaminoglycan (GAG) staining with scale bar on it**


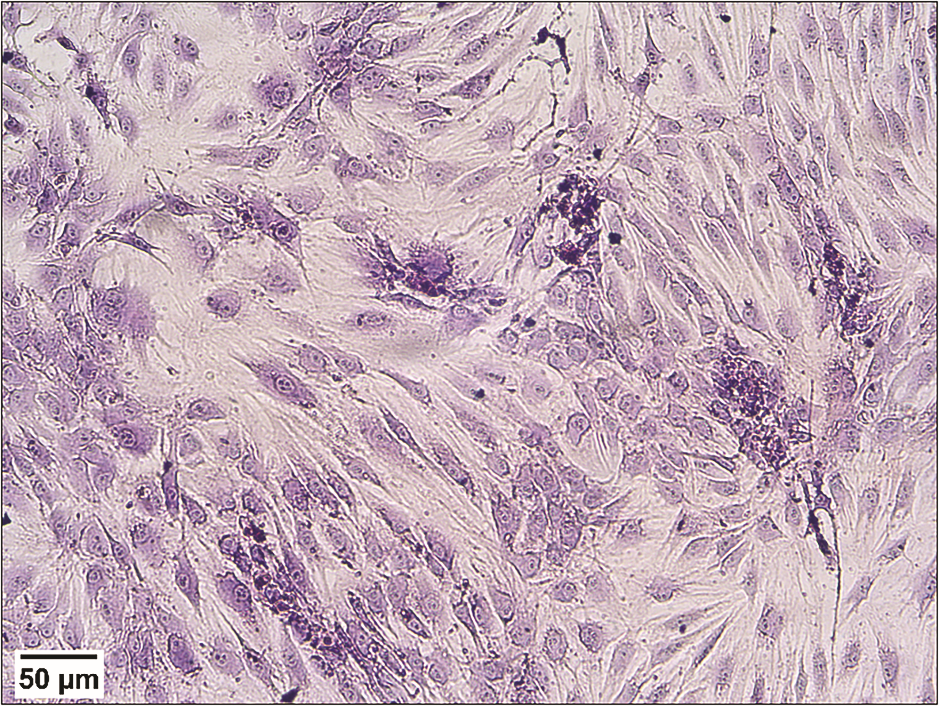


**Figure S3**. Toluidine Blue GAG staining of NC of group 2 with the scale bar shown.

**S4. Variations in the gene expression of key chondrogenic, osteogenic and inflammatory markers as a function of weight and nutraceutical treatment**

**Table S1.** A summary of how gene expression of key chondrogenic, osteogenic and inflammatory markers differed from the negative control as a function of nutraceutical treatment for both groups. Light gray represents a ratio above 1 while dark gray represents a ratio below 1.

| **Nutraceutical**  **/Gene** | **COL2A1** | **COL10A1** | **ACAN** |  | **BMP-2** | **FOXO1** | **SOX-9** |  | **NOS2** | **MMP13** | **TNFIAP6** |
| --- | --- | --- | --- | --- | --- | --- | --- | --- | --- | --- | --- |
| **C group 1** |  |  |  |  |  |  |  |  |  |  |  |
| **C group 2** |  |  |  |  |  |  |  |  |  |  |  |
| **G group 1** |  |  |  |  |  |  |  |  |  |  |  |
| **G group 2** |  |  |  |  |  |  |  |  |  |  |  |
| **Alpha group 1** |  |  |  |  |  |  |  |  |  |  |  |
| **Alpha group 2** |  |  |  |  |  |  |  |  |  |  |  |
| **AA group 1** |  |  |  |  |  |  |  |  |  |  |  |
| **AA group 2** |  |  |  |  |  |  |  |  |  |  |  |
